# Risk factors for *Clostridium difficile* infection in hemato-oncological patients: A case control study in 144 patients

**DOI:** 10.1038/srep31498

**Published:** 2016-08-11

**Authors:** Thorsten Fuereder, Danjel Koni, Andreas Gleiss, Michael Kundi, Athanasios Makristathis, Christoph Zielinski, Christoph Steininger

**Affiliations:** 1 Department of Internal Medicine I, Medical University of Vienna, Währinger Gürtel 18-20, A-1090 Vienna, Austria; 2 Center for Medical Statistics, Informatics, and Intelligent Systems, Medical University of Vienna, Währinger Gürtel 18-20, A-1090 Vienna, Austria; 3 Institute for Enviromental Health, Medical University of Vienna, Währinger Gürtel 18-20, A-1090 Vienna, Austria; 4 Department of Laboratory Medicine, Medical University of Vienna, Währinger Gürtel 18-20, A-1090 Vienna, Austria

## Abstract

Evidence on risk factors for Clostridium difficile infection (CDI) in hemato-oncologic patients is conflicting. We studied risk factors for CDI in a large, well-characterized cohort of hemato-oncological patients. 144 hemato-oncological patients were identified in this retrospective, single center study with a microbiologically confirmed CDI-associated diarrhea. Patients were compared with 144 age and sex matched hemato-oncologic patients with CDI negative diarrhea. Risk factors such as prior antimicrobial therapy, type of disease, chemotherapy and survival were evaluated. CDI-positive patients received more frequently any antimicrobial agent and antimicrobial combination therapy than CDI-negative patients (79% vs. 67%; OR = 2.26, p = 0.038 and OR = 2.62, p = 0.003, respectively). CDI positive patients were treated more frequently with antimicrobial agents active against *C. difficile* than CDI negative ones (25% vs. 13%; OR = 2.2, p = 0.039). The interval between last chemotherapy and onset of diarrhea was significantly shorter in patients without CDI (median, 17 days vs 36 days; p < 0.001). Our study demonstrates that chemotherapy is not a significant risk factor for CDI but for early onset CDI negative diarrhea. The predominant modifiable risk factor for CDI is in hemato-oncological patients antimicrobial treatment. These findings should be taken into account in the daily clinical practice to avoid CDI associated complications and excess health care costs.

*Clostridium difficile* infections (CDI) increased dramatically in severity and incidence during the past decade. An estimated 450,000 cases of *C. difficile* cases occur annually and the rate of nosocomial *C. difficile*–associated diarrhea increased rapidly between 2000 and 2003 in the United States^1^,^2^. Morbidity and mortality associated with CDI is substantial: CDI represents the leading cause of health care associated diarrhea and has a mortality rate of up to 47%^3^. Severe complications, such as the development of a toxic megacolon requiring surgical treatment and prolonged hospital stay, further add to substantial increases in health care costs, which are estimated at 3 billion $ per year in western countries^4^.

The major risk factor for CDI is treatment with antimicrobial agents such as broad-spectrum beta-lactames, quinolones or clindamycin^5^. The antimicrobial suppression of beneficial resident bowel flora results in a dysbiosis that provides a niche *C. difficile* overgrowth^6^. In the case of infection with pathogenic *C. difficile* strains that secrete enterotoxin A and cytotoxin B, these toxins damage the intestinal mucosa and further propagate the dysbiosis. The clinical correlate of this dysbiosis and intestinal inflammation are severe diarrhea leading in some cases to severe and life-threating complications like toxic megacolon, septic shock, or ileus.

Multiple intrinsic and extrinsic influences other than preceding antimicrobial treatment may cause a dysbiosis and thereby increase the risk for CDI. Higher CDI rates were associated with a prolonged hospital stay with a 50% infection rate in patients staying >4 weeks, advanced age, gastric acid suppression, previous CDI, or comorbidities such as diabetes, inflammatory bowel disease, renal insufficiency, immunosuppressive therapy, or prior gastrointestinal surgery^7^,^8^,^9^,^10^,^11^,^12^. In addition, chemotherapy in hemato-oncologic patients has been reported as independent risk factor for CDI^13^.

The development of accurate stratification models for a reliable assessment of CDI risk, however, has been limited by the complex interaction of potential clinical risk factors. Particularly in hemato-oncological patients who are exposed to multiple, concomitant risk factors, evaluation of risk for CDI is problematic. For example, low intensity chemotherapy, duration of hospital stays, and vancomycin use were identified in a small study as independent risk factors for CDI^11^. Platinum-based combination-chemotherapy in the absence of antimicrobial use was identified in two other, small studies as a potential risk factor for CDI^14^,^15^. Other studies reported exposure to other chemotherapeutics such as methotrexate, cisplatin, bleomycin, vinblastine, 5-fluorouracil, cyclophosphamide, doxorubicin, etoposide, paclitaxeland cytarabine as risk for CDI^13^,^16^,^17^. In contrast, more recent studies found similar incidence rates of CDI in chemotherapy patients as in control patients when controlling for the potential confounder of previous antimicrobial therapy^18^,^19^,^20^. Nevertheless, all previous studies were limited by small sample sizes (<40 patients) and frequently by the lack of control cohorts. Identification of robust risk factors for CDI, however, is essential for the development of effective prophylactic strategies.

It was the aim of the current study to investigate risk factors for CDI in a large cohort of hemato-oncological patients with microbiologically confirmed CDI. For this purpose, we compared in one of the largest trials so far clinicopathologic data in 144 CDI-positive hemato-oncological patients with age- and sex-matched CDI-negative hemato-oncological controls. We found that other factors than chemotherapy contribute more significantly to the risk for CDI.

## Patients and Methods

### Data Collection

Patients eligible for this retrospective, single center analysis had a histologically or cytologically confirmed hemato-oncological disease diagnosed between 1^st^ January 2004 and 31^st^ December 2014. Additionally, all cases must have received chemotherapy during the course of their disease and suffered from CDI positive diarrhea in order to be included in this study^21^. Diarrhea had to be either the cause for admission to the hospital or occurred during hospital stay. Prior stem cell transplantation was an exclusion criterion. Patients treated with immunosuppressive agents other than cytotoxic chemotherapy or corticosteroids for anti-tumor treatment were excluded as well. Apart from that four patients were excluded due to daily treatment with targeted therapeutics. Patients from the same database fulfilling these criteria but where diagnosed with CDI-negative diarrhea and have received chemotherapy during the course of their disease served as a control group and were matched by age (max. age difference of 5 years; only for 8 matched pairs the difference was larger than 2 years) and sex with CDI-positive patients for purpose of comparison.

Demographic and clinical data were collected retrospectively from patients’ files and prescription charts and included patients’ age, sex, tumor type, chemotherapy regimen administered, survival data, cause for hospitalization, CDI status and antimicrobial therapies. The study was performed in accordance with the Declaration of Helsinki and good clinical practice guidelines and was approved by the local ethics committee (EK#1432/2014).

### Chemotherapy regimens

Chemotherapeutics were categorized into nine groups in order to evaluate the potential role of distinct chemotherapeutic classes on the risk for CDI. Chemotherapeutic groups comprised platinum drugs (1), anthracyclines (2), 5-fluorouracil (5-FU) and derivatives (3), other antimetabolites than 5-FU (4), taxanes (5), vinca alkaloids (6), topoisomerase inhibitors (7), other alkylating agents (8) and targeted therapies (9). Patients, who did not receive chemotherapy within 30 days prior to CDI positive or negative diarrhea, were classified as having not received chemotherapy recently because of the long interval between chemotherapy and onset of diarrhea.

### Antimicrobial agents

Antimicrobial agents were categorized into β-lactames/cephalosporins (1), glycopeptides (2), carbapenemes (3), quinolones (4), clindamycin (5), and folic acid inhibitors (6). Patients who received antimicrobial agents from other classes were categorized as “others” (7). In addition, antimicrobial agents with *in vitro* activity against *C. difficile* but prescribed within one month before onset of diarrhea were categorized separately (Vancomycin, Teicoplanin, and Metronidazole). Antimicrobial therapy was only considered when administered within one month before onset of diarrhea.

### Microbiological CDI diagnostics

Until February 2007 CDI diagnostics was performed using the Premier toxins A&B enzyme immonoassay as described by the manufacturer (Meridian Bioscience, USA) on a Tecan Minilyser (Tecan Group Ltd., Switzerland). Thereafter all samples were additionally cultured on *C. difficile* agar (bioMérieux, France). In case of borderline or negative EIA results and growth of *C. difficile*, isolates were inoculated to brain heart infusion broth (BHI; Oxoid, United Kingdom) and incubated for 48 h in an anaerobic jar. Culture supernatants were analysed. In March 2009 the Premier toxins A & B EIA was replaced by the Vidas C. difficile toxin A and B assay, which was performed on the mini Vidas as described by the manufacturer (Biomérieux, France). In May 2011 a new testing algorithm has been introduced. Thus, C. diff Chek-60, an EIA for the detection of the *C. difficile* specific antigen glutamate dehydrogenase (GDH), was used as a screening test as described by the manufacturer (TechLab, USA) on the Tecan Minilyser. Positive stool specimens were further analysed by the Cepheid Xpert *C. difficile* real-time PCR assay, which was performed on the Cepheid GeneXpert Dx system following the manufacturers’ instructions (Cepheid, Sunnyvale, CA, USA). A positive PCR result was confirmatory for the presence of a toxigenic strain. Finally, in July 2014 *C. difficile* diagnostics was changed to a 3-test algorithm using the chemiluminescent immunoassay (CLIA) LIAISON *C. difficile* GDH as the initial screening test and the CLIA LIAISON *C. difficile* Toxins A&B as a confirmatory test. Both CLIAs were performed according to the manufacturers’ recommendations (DiaSorin Inc, USA) on a LIAISON Analyzer (DiaSorin). In cases of positive GDH but negative toxin results, specimens were further analysed by the Xpert *C. difficile* PCR. While toxigenic culture is the reference method for CDI detection, Xpert *C. difficile* PCR based assays were described as the most sensitive assays for CDI detection^22^.

### Statistical Analysis

Categorical variables are described by counts and percentages. Continuous variables are described by medians and quartiles due to non-normal distributions. Percentages are compared between matched patient groups (CDI positive vs. negative) using McNemar’s test for variables with two categories and Bowker’s test of symmetry otherwise. The potential influence of various variables on CDI status is investigated in conditional logistic regression models, odds ratios (OR) with 95% confidence intervals are reported. Survival distributions are described as Kaplan-Meier plots, survival at 30 days after CDI diagnosis is compared by McNemar’s test (no patients lost to follow-up within 30 days). Conditional logistic regression is used to adjust this comparison by reason for hospitalization.

A multi-variable conditional logistic regression model was used to adjust the influence of the primary risk factors for each other.

Laboratory parameters at CDI diagnosis are compared using paired t-tests (after log-transformation in the case of leukocytes).

Two-sided p-values ≤0.05 are regarded as statistically significant. The results for the three a priori selected potentially prognostic factors of primary interest (chemo therapy, antibiotics, interval between chemo therapy and diarrhea) are reported irrespective of statistical significance and therefore are not adjusted for multiple testing. Results for secondary hypotheses (detailed chemo therapy and antibiotic variables) are regarded as exploratory; significance after adjustment according to Bonferroni-Holm is indicated in the tables. All computations have been performed using SAS 9.4 (SAS Institute Inc., 2012).

## Results

### Patients

The diagnosis of CDI was established by detection of *C. difficile* toxin or toxigenic *C. difficile* in a stool sample collected from a total of 144 hemato-oncological patients with acute onset diarrhea between 2004 and 2014 at the Medical University of Vienna. Table 1 summarizes relevant demographic data, baseline disease characteristics, antibiotic treatment within 4 weeks before diarrhea, reason for hospitalization and type of chemotherapy within the last 30 days. The CDI-positive patients were mostly elderly with a median age of 67 years (range, 21–90) and 52% of patients were female. In the CDI-positive group, a hemato-oncological disease was as frequently diagnosed as a solid tumor (51% vs. 48%) and no significant difference between the CDI-positive and -negative group could be observed with respect to underlying malignancy (p = 0.342).

Interestingly, diarrhea was the cause for hospitalization only for a minor subset in both CDI-positive and -negative patients (14.1% vs. 9.2%). Admission was most frequently elective and CDI-negative patients were admitted electively more frequently than CDI-positive ones (56% vs. 39.1%; p = 0.010) (Table 1). The second most common reason for admission was in CDI-positive and CDI-negative patients worsening of the general condition due to tumor progression or dyspnea (46.9% vs. 34.8%). Accordingly, diarrhea developed in the majority of patients during the hospital stay.

**Figure 1:**
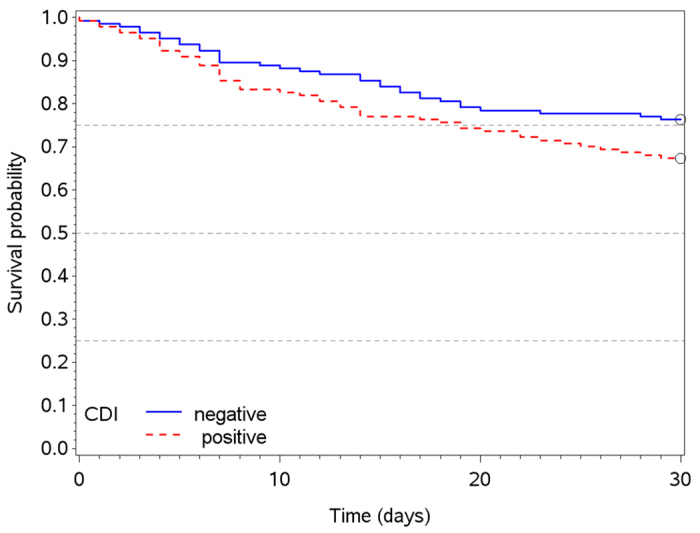
Kaplan meier survival curves of patients up to 30 days after onset of diarrhea in the CDI positive (red) and CDI negative (blue) population.

In total, 83 patients died within 30 days after onset of diarrhea including 47 (33%) and 34 (24%) of CDI positive and –negative patients, respectively. We observed a (not significant) tendency towards shorter survival of patients with CDI positive diarrhea after onset of diarrhea in comparison to CDI negative diarrhea (p = 0.107) (Fig. 1). The patients’ general condition was taken into account by adjusting this comparison for reason of hospitalization (elective chemotherapy vs. other). With reason “elective chemotherapy” there were 9 (18%) vs. 11 (14%) deaths, while for other reasons we observed 34 (44%) vs. 21 (34%). The adjusted survival difference was not statistically significant (p = 0.678).

### Antimicrobial agents

Antimicrobial therapy or prophylaxis is a well-known risk factor for CDI. In the present hemato-oncological cohort, CDI-positive patients had received somewhat more frequently an antimicrobial agent within four weeks prior to onset of diarrhea (79%) as compared to CDI negative patients (67%; OR = 2.26, p = 0.038) (Table 2). Among the various classes of antibiotics, which were administered within four weeks prior to diarrhea, the use of β-lactames/cephalosporins was predominant in CDI positive patients compared to CDI negative ones (53% vs. 42%; OR = 1.88, p = 0.042) (Table 3). The second most common antimicrobial drug were quinolones with a comparable prescription frequency in both groups (39.8% vs. 36.1%; OR = 1.33, p = 0.319) (Table 3). Additionally, more CDI positive patients were treated with folic acid antagonists compared to the CDI negative group (9% vs. 2%; OR = 9.0, p = 0.037). Interestingly, only a minority of patients (4% vs. 2%, OR = 5.29, p = 0.125) received clindamycin, which has been implicated as a risk factor for CDI. Of note, 49% of the patients in the CDI positive arm and 27.6% patients in the CDI negative arm received a combination of antimicrobial agents (OR 2.62, p = 0.003) (Table 3). Remarkably, when antimicrobial agents were categorized for *in vitro* activity against *C. difficile* (i.e. glycopeptides & metronidazole), it became apparent that the 144 CDI positive-patients had received more frequently this group of antimicrobial agents before occurrence of CDI than CDI negative ones (25% vs. 13%; OR = 2.2, p = 0.039) (Table 2).

### Chemotherapeutics and time to diarrhea

**Figure 2:**
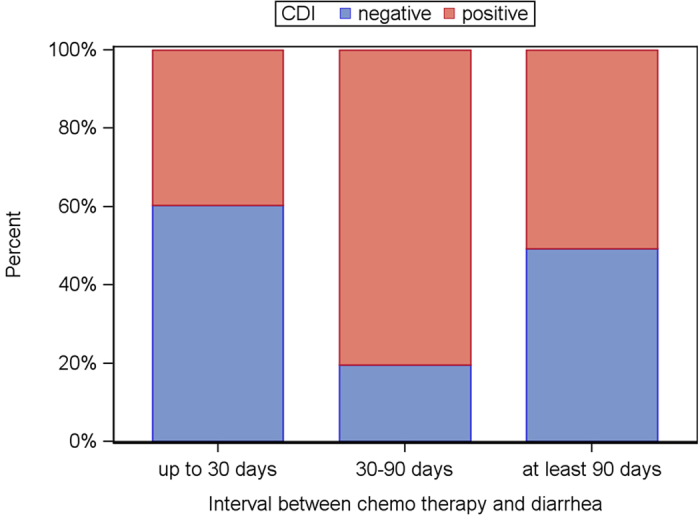
CDI status (blue: negative; red: positive) depending on the interval between chemotherapy and diarrhea.

Since previous studies reported chemotherapy to be an independent risk factor for CDI, we analyzed in a next step if there was an association between chemotherapy and CDI status in our population^13^. The interval between last chemotherapy and onset of diarrhea was significantly shorter in patients without CDI compared to the CDI positive ones (median, 17 days vs. 36 days; p = 0.003) (Fig. 2 and Table 2).

Since distinct chemotherapeutics were reported to change the intestinal microbiome (irinotecan) or are associated specifically with CDI (taxanes), we categorized chemotherapeutics used in the treatment of the present patients into nine different groups as described above. Interestingly, we did not identify chemotherapeutic regimens, which increased the risk for CDI in our population (Table 4).

Of note, elevated leukocyte levels were found in patients with a positive CDI status (median 6.6; IQR: 3.4–11.1) compared to negative ones (median 5.2; IQR: 2.1–9.2; p = 0.008) at the time of CDI diagnosis. No statistically significant difference was observed in hemoglobin or platelet count between the two groups (p = 0.858 and p = 0.559, respectively).

## Discussion

CDI remains a significant clinical and economic burden for health care systems in industrialized countries. In the last couple of years, a changing epidemiology has been observed and CDI has replaced methicillin-resistant *Staphylococcus aureus* as the most common hospital-acquired infection overall^23^. Tremendous progress has been made with respect to identification of risk factors, prognostic biomarkers and development of novel therapies for CDI. Nevertheless, current evidence on risk factors for CDI in hemato-oncologic patients remained conflicting. In this retrospective study-to the best of our knowledge one of the largest ever conducted in this population-we provide compelling evidence that the single most significant risk factor for CDI is previous antimicrobial treatment also in this patient population.

Hematologic malignancies such as lymphomas or multiple myelomas were the first reported to be independent risk factors for CDI based on small case series^11^,^24^,^25^. Following these initial reports, patients with solid tumors such as breast cancer, head and neck cancer and lung cancer were also identified to be at increased risk for CDI^19^,^26^. In the present study, we did not find a significant difference in risk for CDI between patients with hematological or solid malignancy. We also cannot draw conclusions on the risk for CDI associated with the diagnosis of a malignant disease as we did not include healthy individuals as controls. Nevertheless, this question warrants further evaluation as we could confirm that most patients with a malignant disease receive antimicrobial agents and the disease may not be an independent risk factor for CDI.

CDI positive hematologic patients receiving chemotherapy have reportedly similar survival rates compared to immunocompetent patients^18^. Additionally, Bloomfield *et al.* showed in a comprehensive review of the current literature that cancer is not a risk factor for CDI associated mortality^27^. However, these data have to be interpreted with caution: Just a small subset of these trials was primarily done in hemato-oncological patients which results in a small sample-size and subgroup analysis.

The role of chemotherapy exposure as a risk factor for CDI is still unclear. While some studies observed an association between chemotherapy and CDI, more recent studies did not find such a relationship^13^,^16^,^19^. Likewise, our results support the notion that there is no association between a distinct cytotoxic agent or targeted agent and CDI. Interestingly, patients, who received chemotherapy within 30 days before diarrhea, were more likely to suffer from CDI negative disease. Cytotoxic chemotherapy results in mucosal damage which is linked to apoptosis in intestinal crypts and disruption of tight junction proteins within the gut^28^,^29^. These alterations result in post-chemotherapy diarrhea. Moreover, several chemotherapeutic regimens are increasingly recognized as significant modulator of the intestinal microbiome leading ultimately to dysbiosis^30^. Based on these considerations it is tempting to speculate that chemotherapy causes direct intestinal damage resulting in CDI negative diarrhea and in a long term modulation of the intestinal microbiome. This modulation might contribute to the delayed CDI positive diarrhea we observed in our study.

Antimicrobial therapy is a well-known risk factor for CDI in patients without malignancy. In the present evaluation we found that antimicrobial therapy is also a major risk factor for CDI in hemato-oncologic patients (OR = 2.26; CI 1.05–4.88, p = 0.038). Especially patients who received a combination antimicrobial therapy for neutropenic fever were at high risk for CDI positive diarrhea. Remarkably, a considerable number of CDI-positive patients already received an agent with antimicrobial activity against *C. difficile* before onset of diarrhea. This observation emphasizes the notion made more than 20 years ago that every antimicrobial agent may nurture a gastrointestinal dysbiosis that provides a niche for *C. difficile* replication and toxin production^31^. All the more, a rational use of antimicrobial agents has to be advocated in hemato-oncologic patients as this is significant and modifiable risk factor for CDI.

Significant neutropenia in the present CDI positive group, which is a well-known indicator for the severity of CDI, was not associated with higher mortality as reported previously^18^. On the other hand, leukocytosis and renal failure were reported to be a risk factor for the severity of CDI as well in a recent analysis of two randomized trials with 1105 CDI patients^32^. In our analysis, CDI positive patients had higher leukocyte levels compared to the CDI negative group but no difference was detected with respect to serum creatinine levels. However, these findings did also not translate into a significant survival difference between the two groups, although a tendency towards shorter survival was observed for the CDI positive population. However, when adjusting by admission mode, this tendency diminishes, which might reflect a potential bias in our study due to a worse general condition in the patients, who were not admitted to hospital for elective chemotherapy but for diarrhea or other reasons.

The present analysis of 144 CDI positive patients is the largest one reported so far in hemato-oncologic patients. Still, a potential shortcoming of the present study may be the design of a retrospective, case-control study which is by definition prone to information bias and potential confounders. However, in order to conduct prospective or cohort studies for addressing this research question, enormously large and difficult to obtain patient numbers and expensive trials would be necessary to gain information in this special population. The successive development of CDI diagnostics during the study period, which improved the diagnostic accuracy and particularly sensitivity of laboratory methods, may have also biased somewhat the differentiation between CDI-positive and –negative patients with diarrhea. Finally, we have not been able to analyze a potential association of so-called hypervirulent strains (e.g. ribotype 027) with regard to the clinical outcome in CDI positive patients, because ribotype identification is not part of the routine microbiological diagnostics. However, these strains have been associated with more severe disease only in epidemic situations, whereas the present cohort included only sporadic cases of CDI^33^.

In conclusion our study demonstrates that chemotherapy per se is not a risk factor for CDI in hemato-oncological patients. On the contrary, patients, who have received chemotherapy within 30 days before diarrhea, were more likely to suffer from CDI negative diarrhea. Antimicrobial therapy was a major risk factor observed independently from chemotherapy in the present cohort. The use of antimicrobial agents should be careful and rational because this is the single most important modifiable risk factor for CDI in hemato-oncological patients.

## Additional Information

**How to cite this article**: Fuereder, T. *et al.* Risk factors for *Clostridium difficile* infection in hemato-oncological patients: A case control study in 144 patients. *Sci. Rep.* **6**, 31498; doi: 10.1038/srep31498 (2016).

## Figures and Tables

**Table 1:** Patient characteristics.

Patient characteristics	CDI positive (n = 144)	CDI negative (n = 144)
Median age (range)	67 (21–90)	67 (21–89)
Sex		
Male	69 (48%)	69 (48%)
Female	75 (52%)	75 (52%)
Received antimicrobials 4 weeks before diarrhea		
Yes	93 (78.8%)	80 (67.2%)
No	25 (21.2%)	39 (32.8%)
Unknown	26	25
Antimicrobial classes		
Penicillin/Cephalosporins	63 (53.4%)	50 (42%)
Glycopeptides	17 (14.4%)	14 (11.8%)
Carbapenems	19 (16.1%)	15 (12.6%)
Quinolones	47 (39.8%)	43 (36.1%%)
Clindamycin	4 (3.4%)	2 (1.7%)
Metronidazole	15 (12.8%)	6 (5%)
Folic acid antagonists	11 (9.3%)	2 (1.7%)
Other	33 (28%)	16 (13.4%)
Reason for hospitalization		
Elective chemotherapy	50 (39.0%)	79 (56.0%)
Diarrhea	18 (14.1%)	13 (9.2%)
Other (Mainly worsening general condition and dyspnea)	60 (46.9%)	49 (34.8%)
Unknown	16	3
Chemotherapy within 30 days before diarrhea		
Yes	61 (43.9%)	95 (68.3%)
No	78 (56.1%)	44 (31.7%)
Unknown Type	5	5
Platinum drugs	14 (10.1%)	24 (17.3%)
Anthracyclines	17 (12.2%)	31 (22.3%)
5-FU and derivatives	10 (7.2%)	14 (10.1%)
Other antimetabolites	28 (20.1%)	45 (32.4%)
Taxanes	8 (5.8%)	16 (11.5%)
Vinca alkaloids	11 (7.9%)	16 (11.5%)
Topoisomerase inhibitors	16 (11.5%)	25 (18.0%)
Other Alkylating agents	9 (6.5%)	14 (10.1%)
Targeted therapies	26 (18.7%)	25 (18.0%)
Underlying disease		
Hematologic disease	73 (50.7%)	69 (47.9%)
Solid tumor	68 (47.2%)	75 (52.1%)
Both	3 (2.1%)	0 (0%)
AML	22 (15.3%)	29 (20.3%)
AML plus solid tumor	3 (2.1%)	0 (0%)
ALL	5 (3.4%)	7 (4.9%)
Astrocytoma	0 (0%)	1 (0.7%)
Bladder cancer	3 (2.1%)	3 (2.1%)
Breast cancer	12 (8.3%)	9 (6.3%)
Cholangiocellular carcinoma	1 (0.7%)	1 (0.7%)
Cervix carcinoma and endometrial cancer	3 (2.1%)	1 (0.7%)
CLL	7 (4.9%)	1 (0.7%)
CML	0 (0%)	1 (0.7%)
CMML	4 (2.8%)	0 (0%)
Colorectal cancer	6 (4.2%)	11 (7.7%)
CUP	0 (0%)	5 (3.5%)
Head and neck cancer	1 (0.7%)	2 (1.4%)
Lung cancer	12 (8.3%)	6 (4.2%)
Lymphoma	27 (18.8%)	21 (14.7%)
MDS	2 (1.4%)	3 (2.1%)
Mesothelioma	2 (1.4%)	0 (0%)
Myeloma	5 (3.5%)	7 (4.9%)
NET lung, bladder and pancreatic	0 (0%)	5 (3.5%)
Esophagial and gastric cancer	7 (4.9%)	10 (7.0%)
Ovarian cancer	1 (0.7%)	2 (1.4%)
Pancreatic cancer	10 (6.9%)	4 (2.8%)
Prostate cancer	3 (2.1%)	6 (4.2%)
Peritoneal carcinoma	1 (0.7%)	0 (0%)
Renal cell carcinoma	4 (2.8%)	3 (2.1%)
Sarcoma	3 (2.1%)	4 (2.8%)
Seminoma	0 (0%)	1 (0.7%)
Unknown	0	1

Percentages refer to non-missing values; acute myeloid leukemia (AML); acute lymphocytic leukemia (ALL); chronic lymphoid leukemia (CLL); chronic myeloid leukemia (CML); chronic myelomonocytic leukemia (CMML); cancer of unknown primary (CUP); myelodysplatic syndrome (MDS); neuroendocrine tumor (NET).

**Table 2:** Risk factors for CDI.

Risk factor for CDI	Odds ratio from simple models	95% CI	p-value	Odds ratio from multi-variable model	95% CI	p-value
Intervall chemotherapy to diarrhea	OR(30–90 vs. ≤30 days) = 6.09 OR(≥90 vs. ≤30 days) = 1.15	2.56–14.48 0.62–2.15	p < 0.001^a^	OR(30–90 vs. ≤30 days) = 8.64 OR(≥90 vs. ≤30 days) = 1.33	2.48–30.06 0.66–2.67	p = 0.003^a^
Antibiotic therapy within 30 days	OR = 1.92	0.98–3.76	p = 0.056	OR = 2.26	1.05–4.88	p = 0.038

^a^p-value for three-stage variable (≤30/30–90/≥90 days).

**Table 3:** Exploratory analysis of antibiotics as risk factors for CDI.

Risk factor for CDI-	Odds ratio	95% CI	p-value
CDI active antimicrobial therapy	OR = 2.20	1.04–4.65	p = 0.039
Combination of Anitibiotics	OR = 2.62	1.38–4.96	p = 0.003*
β-lactames/cephalosporins	OR = 1.88	1.02–3.44	p = 0.042
quinolones	OR = 1.33	0.76–2.35	p = 0.319
clindamycin	OR = 5.29	0.9–inf**	p = 0.125
folic acid antagonists	OR = 9.00	1.14–71.04	p = 0.037

* significant after Bonferroni-Holm adjustment for multiple testing; ** infinite upper confidence limit, estimated by exact conditional logistic regression due to few Clindamycin patients.

**Table 4:** Exploratory analysis of chemotherapy classes as risk factors for CDI.

Risk factor for CDI-Chemotherapy classes	Odds ratio	95% CI	p-value
Platinum drugs	OR = 0.55	0.27–1.10	p = 0.091
Topoisomerase inhibitors	OR = 0.59	0.30–1.17	p = 0.133
Anthracyclines	OR = 0.52	0.27–1.02	p = 0.056
Taxanes	OR = 0.42	0.15–1.18	p = 0.100
